# Efficacy of Tumor-Targeting *Salmonella* A1-R on a Melanoma Patient-Derived Orthotopic Xenograft (PDOX) Nude-Mouse Model

**DOI:** 10.1371/journal.pone.0160882

**Published:** 2016-08-08

**Authors:** Mako Yamamoto, Ming Zhao, Yukihiko Hiroshima, Yong Zhang, Elizabeth Shurell, Fritz C. Eilber, Michael Bouvet, Makoto Noda, Robert M. Hoffman

**Affiliations:** 1 AntiCancer, Inc., Ostrow Street, San Diego, California, United States of America; 2 Department of Surgery, University of California San Diego, West Arbor Drive, San Diego, California, United States of America; 3 Department of Molecular Oncology, Kyoto University Graduate School of Medicine, Yoshida-Konoe-cho, Sakyo-ku, Kyoto, Japan; 4 Division of Surgical Oncology, University of California Los Angeles, Los Angeles, California, United States of America; Istituto Superiore di Sanità, ITALY

## Abstract

Tumor-targeting *Salmonella enterica* serovar Typhimurium A1-R (*Salmonella* A1-R) had strong efficacy on a melanoma patient-derived orthotopic xenograft (PDOX) nude-mouse model. GFP-expressing *Salmonella* A1-R highly and selectively colonized the PDOX melanoma and significantly suppressed tumor growth (*p* = 0.021). The combination of *Salmonella* A1-R and cisplatinum (CDDP), both at low-dose, also significantly suppressed the growth of the melanoma PDOX (P = 0.001). *Salmonella* A1-R has future clinical potential for combination chemotherapy with CDDP of melanoma, a highly-recalcitrant cancer.

## Introduction

Melanoma is a recalcitrant cancer. When melanoma metastasizes to regional lymph nodes, the 5-year survival rate is 29% and when it metastasizes to organs, the survival rate is 7% [1]. Although recently-developed immunotherapy has extended survival to some extent, the 5-year survival rates have not been significantly increased [2]. Decarbazine and cisplatinum have been used to treat melanoma with limited efficacy [3, 4]. Therefore, more effective approaches to melanoma treatment are needed.

Immunotherapy involving PD-1/PD-L1 blockade has had some success with melanoma but is limited by lack of sufficient tumor infilation of activated lymphocytes to kill the cancer cells within the tumor in the majority of patients tested [5].

Tumor-targeting *Salmonella* A1-R developed by our laboratory is auxotrophic (leucine-arginine dependent) which prevents it from continuously infecting normal tissues. Monotherapy using *Salmonella* A1-R was able to regress or eliminate primary and/or metastatic tumors in models of mouse highly aggressive of prostate [6, 7], breast [8–10], lung [11, 12], pancreatic [13–17], ovarian [18, 19], stomach [20], and cervical cancer [21], as well as sarcoma [22–26] and glioma [27, 28].

Patient-derived orthotopic xenograft (PDOX) models were developed by our laboratory [29, 30]. In the PDOX models, the patient’s tumor is transplanted in the organ of nude or other immunocompetent mice corresponding to its origin and thereby metastasizes such that the tumor mimics the complexity of tumor behavior in patients. Our laboratory has developed PDOX models of all major tumor types including colon [29], pancreatic [31], breast [32], ovarian [33], lung [34], cervical [21], stomach cancer [35], as well as mesothelioma [36] and sarcoma [25, 26, 37, 38].

The aim of the current study was to determine the efficacy of *Salmonella* A1-R on a PDOX model of melanoma compared to and in combination with standard chemotherapy.

## Materials and Methods

### Animal Experiments and Ethics Statement

All animal studies were conducted with an AntiCancer Institutional Animal Care and Use Committee (IACUC) protocol specifically approved for this study and in accordance with the principles and procedures outlined in the National Institutes of Health Guide for the Care and Use of Animals under Assurance Number A3873-1. Athymic nu/nu nude mice (AntiCancer, Inc., San Diego, CA), 4–6 weeks old, were used in this study. Animals were housed in a barrier facility on a high-efficiency particulate arrestance (HEPA)-filtered rack under standard conditions of 12-hour light/dark cycles. The animals were fed an autoclaved laboratory rodent diet (S1 File). In order to minimize any suffering of the animals, anesthesia and analgesics were treated for all surgical procedures. Animals were anesthetized by subcutaneous (s.c.) injection of a 0.02 ml solution of 20 mg/kg ketamine, 15.2 mg/kg xylazine, and 0.48 mg/kg acepromazine maleate. The response of animals during surgery was monitored to ensure adequate depth of anesthesia. Ibuprofen (7.5 mg/kg orally in drinking water every 24 hours for 3 days post-surgery) was used in order to provide analgesia post-operatively in the surgically-treated animals. The animals were carefully observed on a daily basis and would be humanely sacrificed by CO_2_ inhalation if they met the following humane endpoint criteria: prostration, skin lesions, significant bodyweight loss, difficulty breathing, epistaxis, and rotational motion. Individual cages housed animals only in the same treatment group with no more than five mice per cage.

### Melanoma Specimen Collection

The patient had given written informed consent for experimental research on residual tumor tissue available after histopathologic and cytogenetic analyses. The written informed consent document was recorded in a special binder for such documents. The consent procedure was approved by the Institutional Review Board of the UC San Diego Medical Center. This study was also conducted under the approval of the UCSD IRB.

### Patient-Derived Orthotopic Xenograft (PDOX) Melanoma Model

Tumor tissue was obtained from a patient at the time of surgery at the UCSD Medical Center. The harvested tumor was cut into fragments (3-mm^3^) and transplanted into the back skin of five nude mice with two mice transplanted with two tumors. Animals were sacrificed at the end of the experiment. Tumors were harvested and fragments were transplanted to one or two sides of the back skin for the next passage and/or for analysis.

### Histology

Four tumors were harvested from four mice. Harvested tumor samples were fixed with 10% formalin solution, embedded into paraffin and sectioned. Hematoxylin and eosin (H&E) staining was performed with standard protocol. For immunohistochemistry, paraffin-embedded tumor sections were stained with a rabbit anti-human MHC class I antibody (1:100; ab52922, Abcam, Cambridge, MA) and a mouse anti-MHC class I H2 Kd + H2 Dd antibody (1:100; ab24229, Abcam). Immunohistochemistry was performed using anti-rabbit and anti-mouse secondary antibodies and avidin/biotin/horseradish peroxidase complex (Dako Denmark A/S, Glostrup, Denmark) and developed with the DAB kit (BD Biosciences, San Diego, CA) [16]. Five microscopic fields were inspected for each tumor.

### *Salmonella* A1-R and Chemotherapy Drugs

Green fluorescent protein (GFP)-expressing *Salmonella* A1-R bacteria were grown overnight in LB medium and then diluted in 1:10 with LB medium. Bacteria were harvested at late-log phase, washed with phosphate buffered saline (PBS), and diluted in PBS [6–8]. Two weeks after transplantation, bacteria and/or chemotherapy were started. 5-fluorouracil (5-FU, Kyowa Hakko Kirin, Co., Tokyo, Japan) and cisplatinum (CDDP, Nippon Kayaku, Co., Tokyo, Japan) solutions were administered via intraperitoneal (i.p.) injection at a dose of 10 mg/kg (5-FU) and 3 or 5 mg/kg (CDDP) once a week. *Salmonella* A1-R was injected intravenously (i.v.) at a dose of 3 or 5 × 10^7^ CFU/body once a week. The approximate volume of the mass was measured with a caliper twice a week and calculated with the following formula: Tumor volume = 4/3π × (d/2)2 × D/2, where d is the minor tumor axis and D is the major tumor axis. Treatment efficacy was indicated as a ratio of the tumor volume at each time point compared with the tumor volume at the beginning of the treatment. Body weight of the mice was measured on a balance twice a week.

### Detection of GFP-Labeled *Salmonella* A1-R Bacteria in Tumor and Organs

Two days before harvesting tumors and normal organs, mice were injected intravenously with *Salmonella* A1-R (5 × 10^7^ CFU/body). Two days later, PDOX tissues from melanoma tumor and normal organs (liver, spleen and blood) were harvested and weighed. The tumor and organs were minced and diluted in 1:1, 1:10 and 1:100 with 100 μl PBS, respectively. Each dilution (10 μl) was spotted on an LB agar plate containing 50 μg/mL ampicillin and the plates were incubated at 37°C for 24 hours. Relative colony numbers are calculated by actual colony number divided by mg of tissue. One tumor and normal organs from one mouse were harvested and the experiment was repeated three times.

### Imaging

The iBox Imaging System (UVP LLC, Upland, CA) was used for imaging GFP-labeled *S*. *typhimurium* A1-R [39–42]. The BHS System Microscope (Olympus) was used for H&E staining and immunohistochemistry.

### Statistical Analysis

STATA 12.0 SE was used for further data analysis. Time points were chosen a priori: 28 days was selected as a final end point, and 10 days was chosen as a midway representative time point. However, data were collected from 9 time points throughout the 28 days, at regular intervals. A repeated-measures regression model was used to assess the tumor volume of the animals over these regular intervals. The within-subject covariance structure of the data was compound symmetric, therefore we proceeded with repeated measure ANOVA including the repeated option to compute p-values for conservative F-tests. Greenhouse-Geisser correction was performed to correct for any violations of sphericity. The treatment-by-time interaction was significant (p = 0.0000) as were the main-effects for treatment and time (p = 0.0000) for *Salmonella* A1-R, CDDP and *Salmonella* A1-R and CDDP combined. The specific comparisons of interest remained significant within this model. Graph of adjusted predictions of interaction of treatment group and time with 95%CI and graph of mean change in tumor volume over time.

## Results

### Patient-Derived Melanoma Growing Orthotopically in Nude Mice

**Fig 1:**
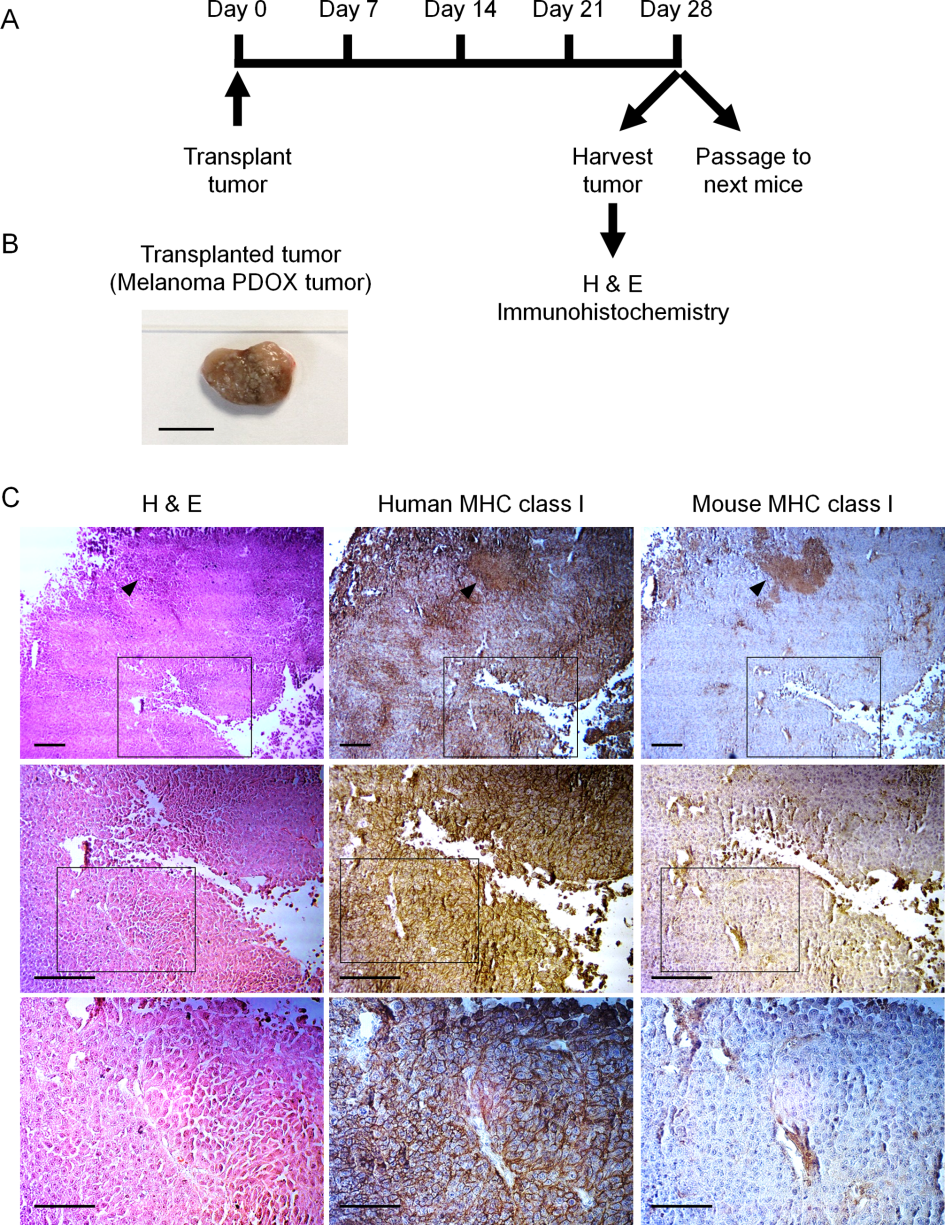
Establishment of a melanoma patient-derived orthotopic xenograft (PDOX) model. **A)** Schematic diagram of the experimental protocol. **B)** Representative cross-sections of transplanted tumor 28 days after transplantation obtained from an orthotopically-transplanted patient’s melanoma. Scale bar: 10 mm. **C)** Immunohistochemical characterization of PDOX melanoma after being grown in nude mice. H&E-stained sections (left column) and immunohistochemistry for human MHC class I (HLA; middle column) and mouse MHC class I (H2 KdtH2 Dd; right column). Strong staining for HLA was observed in the cancer cells (middle column), whereas strong staining for H2 KdtH2 Dd was observed in the stromal cells (right column). Magnified views of boxed region in the upper rows are indicated at the middle rows and magnified views of boxed region in the middle rows are indicated in the lower rows. Black arrowhead indicates necrotic region of the tumor. Scale bars: (top and middle row) 200 μm; (bottom row) 100 μm.

Four weeks after transplantation of the patient melanoma, solid tumors were found growing in the back skin of the nude mice. The tumors were harvested from the mice and used for the next passage and histological analysis (Fig 1A).

To determine whether the grown tumor is completely derived from melanoma patients’ specimen, immunohistochemistry analysis was performed. The melanoma PDOX strongly expressed human MHC class I protein (Fig 1C), whereas cells around blood vessels or stromal cells only reacted with mouse MHC class I antibody (Fig 1C). These data indicate that the growing PDOX tumor was human.

### *Salmonella* A1-R is Highly Effective on the PDOX Melanoma in Nude Mice

**Fig 2:**
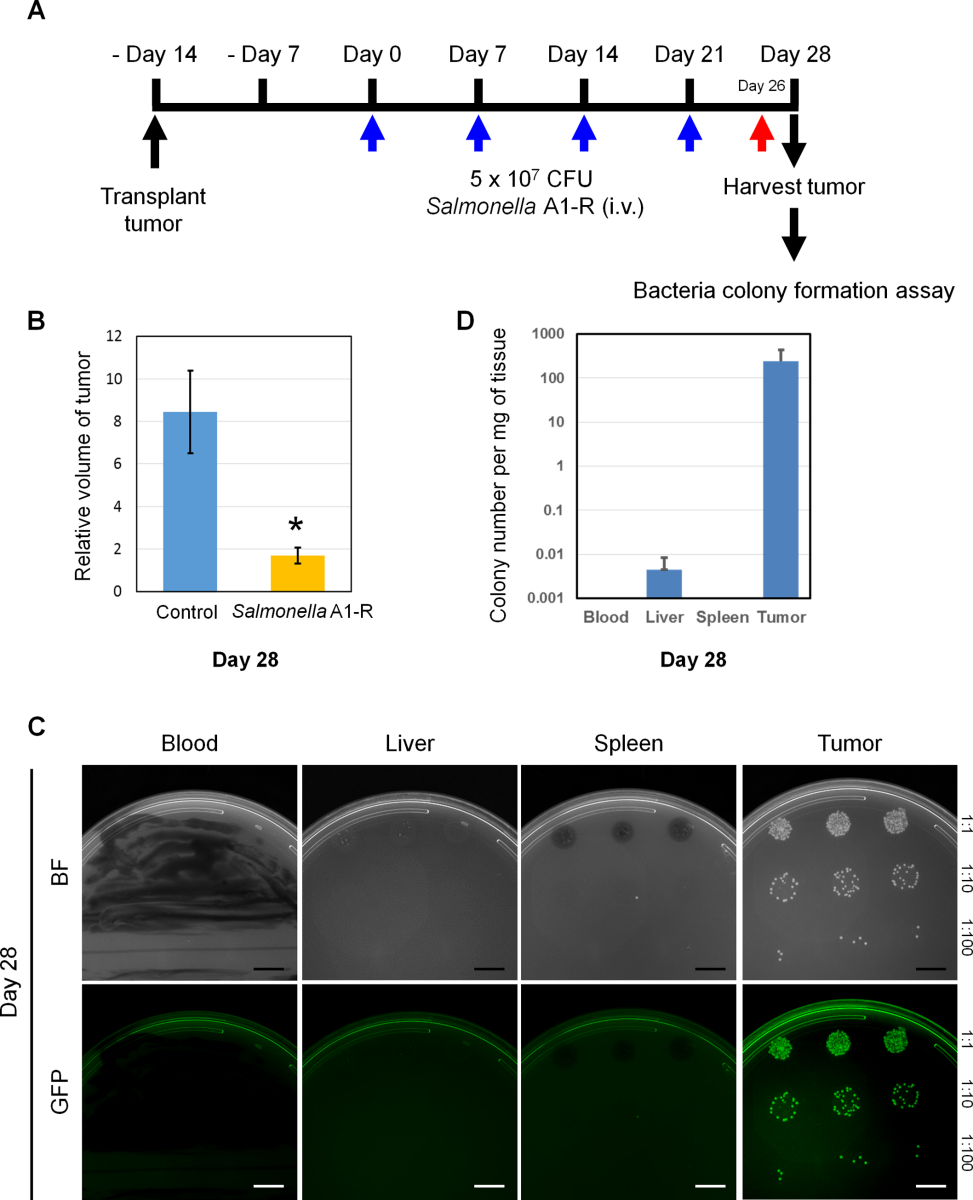
*Salmonella* A1-R targeting and efficacy on the melanoma PDOX model. **A)** Schematic diagram of the experimental protocol. **B)** Efficacy of *Salmonella* A1-R is indicated by the volume ratio of the transplanted tumor at day 28 after injection compared with the tumor at the beginning of the treatment. Tumor size of the *Salmonella* A1-R-treated group was significantly decreased compared with the untreated control group. The values are mean relative tumor volume ± SEM (bars). There were five mice per group. *p < 0.05 compared to the untreated group. **C)** Distribution of GFP-labeled *Salmonella* A1-R in tumor and organs. Representative images of GFP-labeled *Salmonella* A1-R bacteria isolated and cultured from the tumor and the normal organs (blood, liver and spleen) of the mice treated with *Salmonella* A1-R. Fluorescence imaging with the iBox small animal imaging system (UVP LLC). Scale bar: 10 mm. **D)** Colony number of each sample is indicated per mg of harvested tissue. Tissues were collected from three different mice. GFP-labeled *Salmonella* A1-R was clearly detected in the tumor. A small number of GFP-labeled *Salmonella* A1-R was detected in the liver and no GFP-labeled *Salmonella* A1-R was detected in blood and spleen.

*Salmonella* A1-R was administrated intravenously to the melanoma PDOX two weeks after transplantation at a dose of 5 × 10^7^ CFU/body, qW×4. The relative tumor volume on day-28, compared to day-0, of untreated control was 8.46 ± 1.95 and in the *Salmonella* A1-R-treated mice, the tumor volume ratio was 1.68 ± 0.37 (*p* = 0.021) (Fig 2B). There were five mice in each group and the experiment was repeated twice.

Extensive GFP-labeled *Salmonella* A1-R could be isolated from the tumor and could not be isolated from the blood and spleen and only very small amounts could be isolated from the liver (Fig 2C and 2D). These results indicated that *Salmonella* A1-R selectively and effectively colonized and targeted the tumor.

### Efficacy of *Salmonella* A1-R and Chemotherapy on the PDOX Melanoma

**Fig 3:**
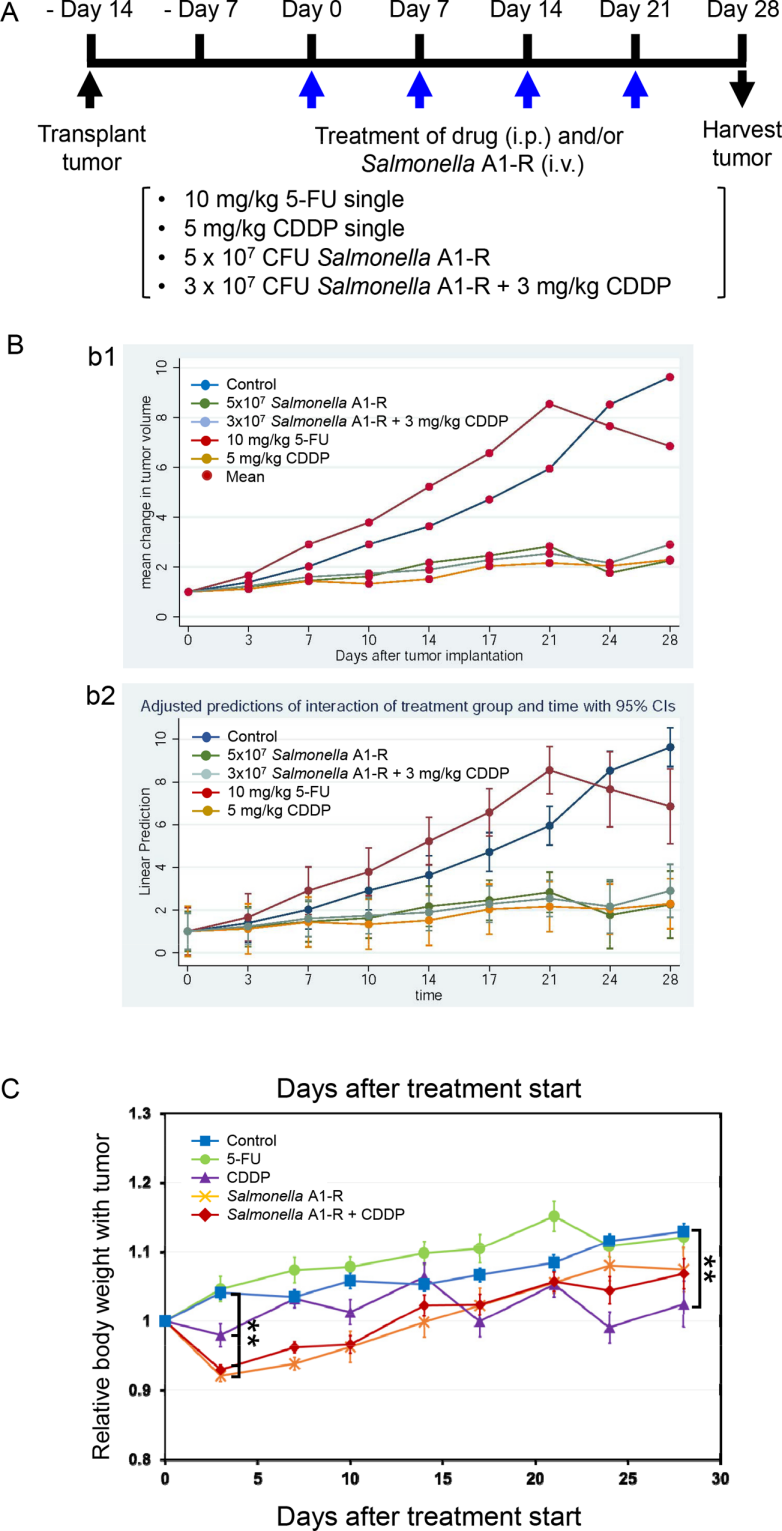
Effect of a tumor-targeting *Salmonella* A1-R and chemotherapy on the melanoma PDOX. **A)** Schematic diagram of the experimental protocol. (1) untreated control (Control); (2) 5-fluorouracil (5-FU; 10 mg/kg, intraperitoneal injection (i.p.), qW×4); (3) cisplatinum (CDDP; 5 mg/kg, i.p., qW×4); (4) *Salmonella* A1-R (5 × 10^7^ CFU/body, intravenously (i.v.), qW×4) and (5) *Salmonella* A1-R (3 × 10^7^ CFU/body, i.v., qW×4) + CDDP (CDDP; 3 mg/kg, i.p., qW×4). **B)** Growth curves of the melanoma PDOX tumor treated with various drugs as described above. B1: Mean change in tumor volume plotted against time; Control, *n* = 9; 5-FU, *n* = 4; CDDP, *n* = 5; *Salmonella* A1-R, *n* = 9; *Salmonella* A1-R + CDDP, *n* = 8. B-2: Data plotted are linear prediction versus time with adjusted predictions of interaction of treatment group and time with 95% Cis. The treatment-by-time interaction was significant (p = 0.0000) as were the main-effects for treatment and time (p = 0.0000) for *Salmonella* A1-R, CDDP and *Salmonella* A1-R and CDDP combined. **C)** Comparison of body weight of nude mice transplanted PDOX tumors after *Salmonella* A1-R and/or chemotherapy. All values represent mean ± SEM; Control, *n* = 8; 5-FU, *n* = 4; CDDP, *n* = 4; *Salmonella* A1-R, *n* = 7; *Salmonella* A1-R + CDDP, *n* = 4. **p < 0.01, compared with the untreated control group.

Two weeks after tumor transplantation, mice were treated with the following groups: (1) untreated control (Control); (2) 5-fluorouracil (5-FU; 10 mg/kg, i.p., qW×4); (3) cisplatinum (CDDP; 5 mg/kg, i.p., qW×4); (4) *Salmonella* A1-R (5 × 10^7^ CFU/body, i.v., qW×4) and (5) *Salmonella* A1-R (3 × 10^7^ CFU/body, i.v., qW×4) + CDDP (CDDP, 3 mg/kg, i.p., qW×4) (Fig 3A).

The relative tumor volume on day 28, compared with day 0, of each group was as follows: (1) untreated control: 9.63 ± 1.37; (2) 5-FU: 6.86 ± 0.52; (3) CDDP: 2.25 + 0.32 (*p* = 0.0001); (4) *Salmonella* A1-R: 2.29 ± 0.35 (*p* = 0.0001); (5) *Salmonella* A1-R + CDDP: 2.90 ± 0.47. The treatment-by-time interaction was significant (p = 0.0000) as were the main-effects for treatment and time (p = 0.0000) for *Salmonella* A1-R, CDDP and *Salmonella* A1-R and CDDP combined. These data suggest that *Salmonella* A1-R and/or CDDP treatment is highly effective in the melanoma PDOX and the efficacy appears from the very early period of the treatment (Fig 3). Regarding the choice of chemotherapy drugs, CDDP was used as a positive control and 5-FU as a negative control in addition to the untreated control, since 5-FU is known not to be effective against melanoma [43].

The relative body weight on day 28 compared with day 0, of each group was as follows: (1) untreated control: 1.13 ± 0.012; (2) 5-FU: 1.12 ± 0.016; (3) CDDP: 1.02 ± 0.033; (4) *Salmonella* A1-R: 1.07 ± 0.032 and (5) *Salmonella* A1-R + CDDP: 1.07 ± 0.022. Only the body weight of CDDP-treated mice was significantly decreased compared with untreated control (*p* = 0.0001) at the end of this experiment (Fig 3C).

## Discussion

*Salmonella* has been previously used for effective cancer therapy of a melanoma and other cell lines [44–58]. The *Salmonella* strain (VNP20009) was attenuated by a lipid A–mutation (msbB), purine auxotrophy (purI) and amino acid auxotrophy [44]. VNP20009 was safely administered to patients in a Phase I clinical trial on patients with metastatic melanoma. However it was poorly colonized in the tumors since it might be over-attenuated [58]. Our results are the first to demonstrate efficacy of *Salmonella* treatment on a patient melanoma tumor models (PDOX).

*Salmonella* A1-R was also previously shown to be active in syngeneic mouse tumor models: we recently determined the efficacy of *Salmonella* A1-R on the Lewis lung (LLC) in C57BL/6 (C57) immunocompetent mice and observed anti-metastatic efficacy [59] as well as against primary tumors [60]. These results suggest that *Salmonella* A1-R is also active in animals with an intact immune system and a syngeneic rather than xenografted tumor. The present and previous results suggest the potential of *Salmonella* A1-R alone or in combination with CDDP to treat melanoma patients in the future.

## Supporting Information

S1 File ARRIVE checklist. (PDF)
